# β-arrestin 2 regulates Aβ generation and γ-secretase activity in Alzheimer’s disease

**DOI:** 10.1186/1750-1326-8-S1-P41

**Published:** 2013-09-13

**Authors:** Amantha Thathiah, Katrien Horre, An Snellinx, Elke Vandewyer, Yunhong Huang, Marta Ciesielska, Gerdien De Kloe, Sebastian Munck, Bart De Strooper

**Affiliations:** 1 VIB Center for the Biology of Disease, Leuven, Belgium; 2 Center for Human Genetics, University of Leuven (KU Leuven), Leuven, Beigium; 3 Neuroscience Medicinal Chemistry, Janssen Research and Development, a Division of Janssen Pharmaceutica NV, Beerse, Belgium

## 

Deficits in several neurotransmitter systems are characteristic features of the brains of AD patients. The majority of neurotransmitters communicate information to cells via G protein-coupled receptors (GPCRs) or 7-transmembrane receptors (7TMRs). It has recently been appreciated that a small family of multifunctional GPCR regulatory known as the β-arrestins which play an almost universal role in facilitating traditional GPCR desensitization, are also capable of initiating distinct signals in their own right, conveying receptor subtype-specific signaling events. These signals are often both spatially and temporally distinct, and result in unique cellular and physiological or pathophysiological consequences. As mediators of GPCR desensitization, trafficking and cell signaling, the β-arrestins provide a putative basis to understand GPCR dysfunction in AD. Here, we report that β-arrestin 2 levels are elevated in two independent cohorts of patients with AD. Genetic deletion of *Arrb2* (β-arrestin 2) reduces accumulation of the amyloid-β (Aβ) peptide in an AD mouse model. Consistent with these observations, endogenous murine Aβ generation is also reduced in *Arrb2*^-/-^ mice. Elucidation of the mechanism of the β-arrestin 2-mediated effect on Aβ levels indicates that recruitment of β-arrestin 2 to two GPCRs implicated in the pathogenesis of AD, GPR3 and the β_2_-adrenergic receptor (β_2_-AR), is required for the promotion of Aβ release. Collectively, these studies identify β-arrestin 2 as a novel avenue for targeting amyloid pathology and GPCR dysfunction in AD.

